# Mitigating the Undesirable Chemical Reaction between Organic Molecules for Highly Efficient Flexible Organic Photovoltaics

**DOI:** 10.1002/advs.202100865

**Published:** 2021-05-07

**Authors:** Adi Prasetio, Muhammad Jahandar, Soyeon Kim, Jinhee Heo, Yong Hyun Kim, Dong Chan Lim

**Affiliations:** ^1^ Surface Materials Division Korea Institute of Materials Science (KIMS) Changwon‐daero 797 Changwon 51508 Republic of Korea; ^2^ Department of Physics Pukyong National University Yongso‐ro 45 Busan 48513 Republic of Korea; ^3^ Department of Display Engineering Pukyong National University Yongso‐ro 45 Busan 48513 Republic of Korea

**Keywords:** cathode interfacial layers, interfacial modification, nonfullerene acceptors, organic photovoltaics, solar cells

## Abstract

Organic photovoltaics (OPVs) with nonfullerene acceptors (NFAs) feature excellent device performance and device stability. However, they are facing problems when the amine‐rich polyelectrolytes are used as cathode interfacial layers. In this work, a small molecule, ethanedithiol (EDT) at the polyethyleneimine ethoxylated (PEIE)/active layer interface is inserted for mitigating the undesirable reaction between amine‐rich groups and electron‐acceptor moieties in NFA. The main role of EDT is to passivate the PEIE surface and prevent electron flow to NFA and the unwanted reaction can be mitigated. It improves the performance of OPV devices by reducing the work function, decreasing trap‐assisted recombination, and improving electron‐mobility. As a result, the flexible device with the PEIE interfacial layer with a power conversion efficiency (PCE) of 7.20% can be improved to 10.11% after the inclusion of EDT. Moreover, EDT‐modified device can retain 98.18% after it is bent for 200 cycles and can maintain 80.83% of its initial PCE under continuous light illuminated in ambient conditions without any encapsulation. Based on these findings, the proposed strategy constitutes a crucial step toward highly efficient flexible OPVs.

## Graphical abstract

High‐performance nonfullerene‐based flexible organic solar cells are achieved by the small‐molecule‐driven interfacial modification. It can prevent the undesirable chemical reaction between polyelectrolyte‐based interfacial layer and photoactive layer by controlling electron flow direction. This strategy can be used regardless of the type of nonfullerene acceptors in flexible organic photovoltaic technology.

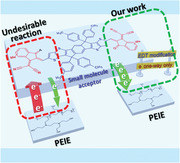

## 1 Introduction

Bulk‐heterojunction (BHJ) organic photovoltaics (OPVs) offer advantages of a lightweight, large‐scale fabrication capability, and superior mechanical flexibility.^[^ ^1^, ^2^, ^3^, ^4^, ^5^ ^]^ These days, the nonfullerene acceptor (NFA) replaces the fullerene derivatives because of their superiority. Compared to the fullerene acceptor, the NFA has numerous advantages, such as tunable bandgap that can broaden the absorption in the near‐infrared region, tunable crystallinity, and planarity that can improve the morphology of the active layer.^[^ ^6^, ^7^ ^]^ Moreover, in the NFA, the required energy offset is ≈0 eV, which significantly reduces the energy loss. The recent reported highest power conversion efficiency (PCE) is 18.22% based on single‐junction NFA.^[^ ^8^, ^9^, ^10^ ^]^ Howbeit, the development of the NF solar cells on flexible substrates is lagging compared to the rigid substrates. The high‐temperature processed and rigid cathode interfacial layers (CILs) retard the development of the NF flexible OPVs. The usage of metal oxides is refrained, for instance, the zinc oxide (ZnO) has photocatalytic activity on the molecular structure of NFA degrading the photostability under light illumination, while the titanium dioxide (TiO_2_) has the light‐soaking effect and high‐temperature processing which is not suitable for flexible devices.^[^ ^3^, ^11^, ^12^, ^13^, ^14^ ^]^

Instead of metal oxides, the nonconjugated polyelectrolytes such as polyethyleneimine (PEI) and polyethyleneimine ethoxylated (PEIE) could be more suitable for flexible devices due to their good mechanical flexibility and low‐processing temperature.^[^ ^15^, ^16^, ^17^ ^]^ In fullerene‐based organic solar cells, the PEIE possesses superior stability and performance compared to the other conjugated polyelectrolytes and metal oxides.^[^ ^17^ ^]^ However, the usage of PEIE or PEI in the OPV devices with an NFA system drastically declines the performance and stability. Unlike the fullerene, the NFA has acceptor‐donor‐acceptor (A‐D‐A) moieties for creating intramolecular charge transport and broadening the light absorption spectrum. For illustrating, the high‐performance ITIC families have an indacenodithieno[3,2‐*b*]thiophene (IDTT) group as an electron donor and two 2‐(3‐oxo‐2,3‐dihydroinden‐1‐ylidene) malononitrile (INCN) groups as electron acceptors.^[^ ^18^, ^19^, ^20^ ^]^ Despite A‐D‐A configuration exhibits outstanding photovoltaic performance, the intramolecular structures of INCN electron‐acceptor are easily attacked by the electron‐rich amine groups of PEIE or PEI acting as nucleophiles with strong electron‐donating properties. Thus, the intramolecular charge transport of the A‐D‐A configuration is retarded in mild cases or even interrupted in severe cases. Moreover, it could also generate increased trap states or electronic disorders in the BHJ.^[^ ^20^, ^21^ ^]^ Therefore, it is a formidable challenge to prevent the reaction between PEIE or PEI with the NFA. A few attempts have been performed to resolve this problem. For instance, Zhou et al. deactivated the reaction on the NFA by adding a proton to the PEIE dissolved in an aqueous solution and acidic aqueous solution.^[^ ^3^ ^]^ In another study, they provided an acidic environment for the acceptor by directly adding H^+^ originated from acetic acid to the active layer solution.^[^ ^20^ ^]^ However, the protonation of PEIE could lessen its functionality to reduce work function (WF) due to the excess protonated‐amines and the acidic environment is not desirable because it could decrease device stability.^[^ ^15^, ^17^ ^]^

**Figure 1:**
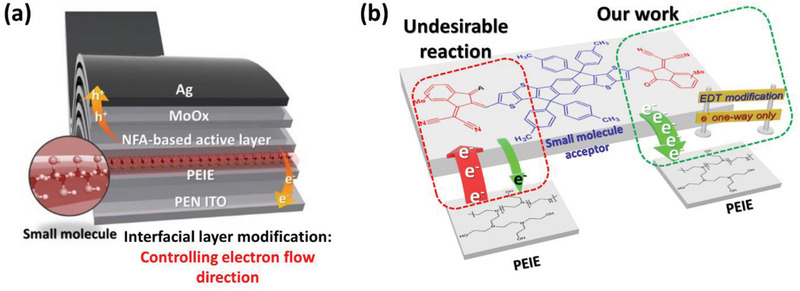
a) Inserting the small molecule at CIL/active layer interface to control electron flow direction at CIL/active layer interface. b) The schematic of our strategy to mitigate reaction occurred in NF acceptor when contact with amines or hydroxyl groups in interfacial layers.

To overcome the mentioned problems, we demonstrated a novel method to mitigate the reaction between PEIE and NFA by introducing the ethanedithiol (EDT) at the PEIE/active layer interface without sacrificing device stability. The mechanism of the reported strategies mentioned before is shown in Figure S1 in the Supporting Information, while our proposed study is shown in **Figure** **1**. The mechanism of mitigating reaction between PEIE and NFA in this report is systematically studied by using conductive‐atomic force microscopy (C‐AFM), absorbance spectroscopy, and Raman spectroscopy. According to our observations, the PEIE reacts with NFA by attacking the C=O moiety that disturbs intramolecular charge transport of A‐D‐A configuration. The electrophile of C=O and dicyano moieties mainly affected the electron donation from electron‐rich amine groups of PEIE. After the inclusion of EDT small molecule, the electron donation from the lone pair nitrogen atoms in PEIE to INCN electron‐acceptor can be suppressed. As a result, the reaction that occurred on the C=O and dicyano moieties could be hindered and the charge transport is not interrupted. Moreover, the PEIE with EDT exhibits lower WF, lower trap‐assisted recombination, and higher electron mobility compared to the pristine PEIE counterparts. Consequently, a significant performance enhancement was obtained for the rigid PEIE‐EDT‐based devices with the power conversion efficiency (PCE) of 12.06% which is higher than those of pristine PEIE with a PCE of only 9.60%. Besides, the PCE of 10.11% was obtained on PEIE‐EDT‐based flexible devices which are superior to PEIE‐based flexible devices (PCE: 7.20%). The device performance improvement was mainly originated from the improved open‐circuit voltage (*V*_OC_) and fill‐factor (FF) of PEIE‐EDT‐based devices. Moreover, the major stability issues such as mechanical and photoinduced degradation under continuous light illumination were also improved in the PEIE‐EDT‐based devices. Finally, to check the universality of our strategy and practical indoor applications, combining different types of photoactive polymers with EDT on flexible devices are employed and tested under low‐light illumination, respectively.

## 2 Results and Discussions

### 2.1 Analysis of Compositional and Surface Properties of Ethanedithiol‐Modified PEIE

**Figure 2:**
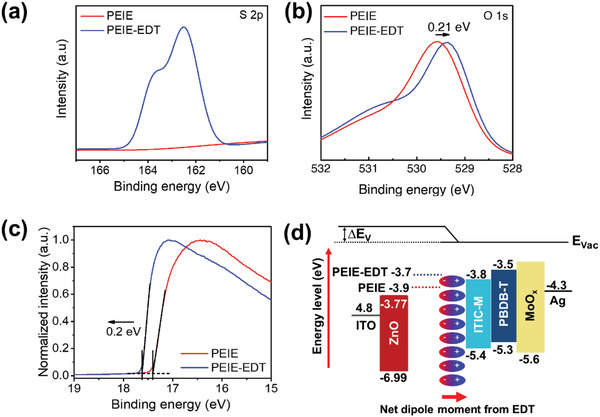
XPS spectra of a) S 2p, and b) O 1s; c) the UPS spectra of PEIE and PEIE‐EDT thin films on ITO glass substrates; d) energy level diagram of inverted OPV with the structure of ITO/CILs/PBDB‐T:ITIC‐M:MoO*_x_*/Ag.

The coverage of the EDT on the PEIE was confirmed by X‐ray photoelectron spectroscopy (XPS) analysis. The wide‐scan XPS spectra are shown in Figure S2 in the Supporting Information and the detailed XPS analysis for C, N, O, and S elements is shown in Figure S3 in the Supporting Information. The S 2p peak appeared at around 162.5 eV after EDT treatment, confirming the formation of thiol on the PEIE surface (**Figure** **2**a). According to the deconvolution of the XPS spectra of S 2p (Figure S3a, Supporting Information), the peak at a binding energy of 162.5 and 163.8 eV, respectively, represents the bonded‐thiol on the PEIE surface and unbounded‐thiol.^[^ ^22^, ^23^ ^]^ It can be assumed that the EDT formation on the PEIE is via chemisorption which was proven by the shifting of the O 1s peak (Figure 2b) to higher binding energy attributed to hydroxyl groups of PEIE which is noticeably decreased after EDT treatment confirming that the thiols were bonded to the —OH of PEIE.^[^ ^24^ ^]^ We suggest that in the case of the O—H and S—H hydrogen bonding, the major component of its interaction is dispersive. The O—H and S—H can be attracted to each other via Keesom forces. The shifting of the peaks to lower energy is due to the lower electronegativity of thiol's elements.

It is well known that the interface modification between the interfacial layer and polymer active layer can affect not only the optical and electrical properties but also the morphology of the interlayer itself and even the active layer. After the modification of the PEIE using EDT, in this study, there are no significant changes observed on the optical and morphology properties (Figures S4 and S5, Supporting Information). However, as shown in Figure S6 in the Supporting Information, the hydrophobicity is improved, enabling better wetting by chlorobenzene solvent that is used for the PBDB‐T:ITIC‐M active layer. The better wetting at CIL/active layer interface could lead to improved active layer film formation and donor/acceptor phase morphology.^[^ ^25^, ^26^ ^]^

**Figure 3:**
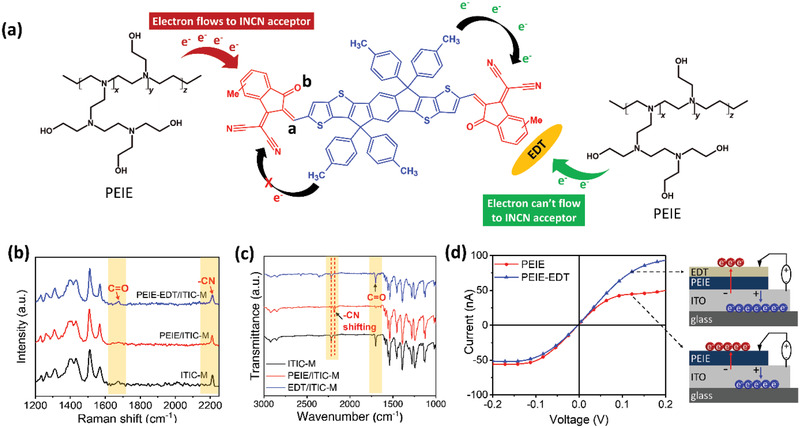
a) Mechanism of the mitigating reaction between PEIE and ITIC‐M using EDT. The marked “a” refers to C=C linkage and “b” refers to C=O moiety. b) Raman spectra of ITIC‐M on the PEIE and PEIE‐EDT CILs. c) The FTIR of ITIC‐M exposed to PEIE and EDT; d) current–voltage curve of PEIE and PEIE‐EDT measured using C‐AFM with the schematic of the electron transport under forward and reverse bias.

The effect of intramolecular interaction caused by the EDT versus NFA will be further discussed. On the other hand, the PEIE modification by EDT induced an interface dipole between PEIE and the active layer and it can strongly affect the charge transport property, results in enhanced device performance, PCE, and photostability as well as mechanical stability of the flexible devices. As shown in Figure 2c, ultraviolet photoelectron spectroscopy (UPS) measurement was implemented to investigate the effect of EDT treatment on WF. The WF of PEIE, approximated from the secondary electron cut‐off region, is shifted from 3.9 to 3.7 eV after EDT treatment confirming the dipole effect. The energy‐band diagram of PEIE and PEIE‐EDT in an inverted OPV device with a structure of indium tin oxide (ITO)/CILs/active layer/MoO*_x_*/Ag is shown in Figure 2d. The change of surface potential varies linearly with the nature of electron‐withdrawing and ‐donating of modifier molecule. The strong electron‐donating characteristic can reduce the surface potential and work function of the semiconductor and which is in line with the result of C‐AFM in **Figure** **3**d. Using C‐AFM, one can simultaneously measure topography and current flow characteristics like distribution, conductivity, and flow direction over the surface. The asymmetric electron flow direction, from PEIE to ITO electrode, is observed dominantly after the EDT modification, which well represents the work function‐based energy level diagram.

### 2.2 Analysis of Interaction Mechanism between PEIE‐EDT and NFA

The chemical structure of materials used in this study is shown in Figure S7 in the Supporting Information and the mechanism of the interaction between PEIE‐EDT and NFA is shown in Figure 3a. PBDB‐T:ITIC‐M and PEIE were used to form inverted BHJ solar cell devices. Figure S17a in the Supporting Information shows the absorption spectra of ITIC‐M NFA film with different CILs. The ITIC‐M with ZnO CIL shows the absorption at the range of 400–800 nm with the peak at ≈700 nm due to its high‐energy *π*–*π* stacking transition.^[^ ^21^ ^]^ For the PEIE CIL, the absorption peak at ≈700 nm drastically decreases and the new absorption appears in the region close to the UV region. Moreover, the change in the UV‐vis absorbance is also found for the ITIC‐M exposed to PEI (Figure S18b, Supporting Information). We also found that the reaction between PEIE/PEI and ITIC‐M is time‐dependent. As the exposure time increases, the reaction becomes more severe which is proven by more significant changes in UV‐vis spectra (Figure S18a,b, Supporting Information).^[^ ^21^ ^]^ The electron‐rich amine groups in PEIE/PEI CIL are working as nucleophiles and react with the C=O and —CN moieties, “poisoning” the ITIC‐M structure, cause the breaking up the original electronic structure and intramolecular electron transfer from the donor IDTT to the broken electron‐accepting INCN end group in the ITIC‐M.^[^ ^3^, ^20^, ^21^ ^]^

To further investigate the reaction between PEIE CIL and ITIC‐M NFA, the Raman spectroscopy was performed and the resulted spectra were shown in Figure 3b. It shows that the amine groups of PEIE attack the carbonyl group C=O in INCN moiety which is proven by the vanishing of a peak located at 1675 cm^−1^ (Figure S8a, Supporting Information). Besides, the peak at 2213 cm^−1^ attributed to —CN bond was also found shifting to lower wavenumber as shown in Figure S8b in the Supporting Information. These results are further confirmed by the Fourier‐transform infrared (FTIR) spectra (Figure 3c). FTIR spectra show that the peak at 1702 cm^−1^ corresponds to C=O is seemly disappeared (Figure S8c, Supporting Information) and the peak at 2218 cm^−1^ attributed to the —CN peak shifts to lower wavenumber (Figure S8d, Supporting Information). The FTIR measurement was also performed for examining the reaction between ITIC‐M and PEI. The FTIR spectra (Figure S19, Supporting Information) of ITIC‐M exposed to PEI show a distinct change in the spectra which is similar to those of PEIE. These results are in good agreement with the previously reported results.^[^ ^21^ ^]^ We further performed the ^1^H NMR study to investigate the in‐depth mechanism of the reaction between PEIE and ITIC‐M. We also included the ITIC‐M/PEI and ITIC‐M/EDT spectra to support the result. As we can see in Figure S8f in the Supporting Information, the spectra of ITIC‐M mixed with PEI and PEIE show different peaks in the aromatic region compared to pure ITIC‐M which depicted that a chemical reaction might be happening between ITIC‐M and PEI/PEIE. The PEI or PEIE reacts with the C=C linkage moiety in PEIE as illustrated in Figure S8e in the Supporting Information via Michael addition reaction that destroyed the chemical and electronic structure of ITIC‐M.^[^ ^3^, ^27^, ^28^ ^]^ In contrast, ^1^H NMR spectra of ITIC‐M and EDT mixture are almost similar to the pure ITIC‐M spectra which confirm there is no reaction between ITIC‐M and EDT. Since the polymer amines are too complicated to test by NMR, the final product formed after the reaction of PEI or PEIE with ITIC‐M could not be perfectly characterized. The amines in PEIE may take up proton from thiols. In other words, the thiol group in EDT can mitigate the reaction with a functional group (marked “a” in Figure S8e, Supporting Information) in ITIC‐M by interaction with PEIE.^[^ ^29^ ^]^

However, the attacked ITIC‐M can be drastically suppressed by the inclusion of EDT on the surface of PEIE. As a proof, there are no noticeable changes in UV‐vis absorbance (Figure S17a, Supporting Information) and Raman spectra (Figure 3b) between the ITIC‐M layer only and the ITIC‐M layer with PEIE‐EDT interlayer. To confirm there is no reaction between ITIC‐M and EDT, we analyzed the UV‐vis absorbance (Figure S17b, Supporting Information) of ITIC‐M and ITIC‐M purposely mixed with PEIE and EDT. The results show that there is no reaction occurred between the EDT with either C=O or —CN groups.

Figure 3d shows the current–voltage curve of PEIE and PEIE‐EDT measured by using C‐AFM under reverse and forward bias and the current distribution mapping is shown in Figure S9 in the Supporting Information. Under reverse bias, the PEIE‐EDT shows a lower current than those of PEIE pristine which indicating suppressed electron flow toward the PEIE direction. The lower current flow toward the PEIE direction can prevent electron donation from the PEIE to ITIC‐M acceptor.

### 2.3 Device Performance of Rigid and Flexible OPV Devices

**Figure 4:**
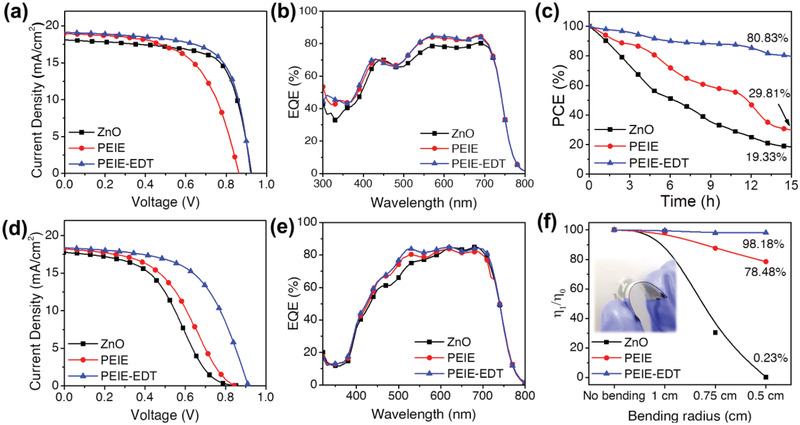
a) Current density–voltage (*J*–*V*) characteristics, b) EQE spectra of rigid PBDB‐T:ITIC‐M OPV devices; c) photostability of rigid PBDB‐T:‐ITIC‐M OPV devices under 1 sun light illumination continuously for 15 h; d) current density–voltage (*J*–*V*) characteristics, e) EQE spectra of flexible PBDB‐T:ITIC‐M OPV devices; f) bending‐stability of flexible PBDB‐T:ITIC‐M OPV devices with various bending radius which bent for 200 cycles.

To understand the effect of the alkyl chains and the photovoltaic performance, we perform a preliminary study by comparing the EDT and hexanedithiol (HDT). As a result, the EDT has higher efficiency compared to the HDT due to its higher FF and *J*_SC_ as shown in Figure S10 and Table S1 in the Supporting Information, which might be associated with the lower conductance of longer alkyl chain HDT. Moreover, the longer alkyl chain between the active layer and the cathode could disturb the ability of electrons for tunneling.^[^ ^30^ ^]^ The detailed device performance for both rigid and flexible OPV devices with ZnO, PEIE, and PEIE‐EDT CILs is summarized in **Table** **1**. The device statistics of PCE, *V*_OC_, *J*_SC_, and FF for rigid and flexible devices are shown in Figures S11 and S12 in the Supporting Information, respectively. **Figure** **4**a shows the *J*–*V* characteristics of the rigid OPV devices containing the ZnO, PEIE, and PEIE‐EDT CILs. The PCE of the ZnO‐based device was 11.76% with a *V*_OC_ of 0.93 V, *J*_SC_ of 18.09 mA cm^−2^, and FF of 0.70. Meanwhile, the PEIE‐based device shows a poor PCE of 9.60% with a *V*_OC_ of 0.86 V, *J*_SC_ of 18.92 mA cm^−2^, and FF of 0.59. It shows that the lower performance of the PEIE‐based device can be mainly attributed to the low *V*_OC_ and FF. Nevertheless, the device performance was noticeably improved by the inclusion of the EDT between PEIE and the active layer. The PCE of the device with EDT‐modified PEIE was 12.06%. The device parameters *V*_OC_, FF, and *J*_SC_ are simultaneously improved to 0.92 V, 0.689, and 19.16 mA cm^−2^. As shown in Figure 4b, the external quantum efficiency (EQE) value of the PEIE‐EDT‐based device shows a slightly higher value compared to those of ZnO and PEIE throughout the visible light region. Notably, the PEIE‐EDT‐based device exhibits lower *V*_OC_ loss (0.69 V) according to the equation *E*_g_/*q*−*V*_OC_, where the *E*_g_ (1.61 eV) is the energy gap from the Tauq plot (Figure S13, Supporting Information) and *q* is the elementary charge.^[^ ^31^, ^32^ ^]^ We also fabricated OPV devices with PEI as CILs, resulting in poor device performance. However, after the inclusion of EDT on PEI, the overall performance was significantly improved (Figure S20, Supporting Information). The photovoltaic properties are summarized in Table S7 in the Supporting Information. The photostability of rigid OPV devices shown in Figure 4c was measured under continuous light illumination for 15 h in an ambient environment. After 15 h, the PEIE‐EDT device retained 80.83% of its initial PCE, whereas the ZnO and PEIE devices lost a lot of their performance. The lower photostability of the ZnO‐based device is related to the photocatalytic activity of ZnO on NFA.^[^ ^13^ ^]^

**Table 1:** The detailed device performance parameters of the flexible OPVs based on PBDB‐T:ITIC‐M photoactive layer with ZnO, PEIE, and PEIE‐EDT CILs

Type	CILs	PCE [%] (PCE_ave_)^a^	*V*_OC_ [V]	*J*_SC_ [mA cm^−2^]	FF	*R* to *F* ratio [%]^b^
Rigid	ZnO	11.76 (11.29 ± 0.20)	0.93 (0.91 ± 0.01)	18.09 (17.42 ± 0.35)	0.70 (0.70 ± 0.01)	
	PEIE	9.60 (9.30 ± 0.39)	0.86 (0.83 ± 0.01)	18.92 (18.17 ± 0.43)	0.59 (0.61 ± 0.02)	
	PEIE‐EDT	12.06 (11.67 ± 0.15)	0.92 (0.91 ± 0.01)	19.16 (18.55 ± 0.07)	0.69 (0.69 ± 0.02)	
Flexible	ZnO	6.42 (5.95 ± 0.44)	0.87 (0.85 ± 0.02)	17.75 (17.70 ± 0.14)	0.42 (0.41 ± 0.03)	34.59
	PEIE	7.20 (6.69 ± 0.78)	0.84 (0.82 ± 0.01)	18.21 (17.84 ± 0.32)	0.49 (0.46 ± 0.04)	75.00
	PEIE‐EDT	10.11 (9.91 ± 0.22)	0.87 (0.86 ± 0.01)	18.44 (18.22 ± 0.22)	0.63 (0.63 ± 0.01)	83.83

^a)^ The values in parentheses stand for the average performance from over 12 devices

^b)^ Calculated from (PCE_Flexible_/PCE_Rigid_)*100%.

To find out the flexible capability of flexible OPVs with various CILs, we fabricated inverted structure flexible devices with PBDB‐T:ITIC‐M active layer. The *J*–*V* curve of the flexible OPV devices with various CILs is shown in Figure 4d. The champion flexible PEIE‐EDT‐based device achieved a PCE of 10.11% with a *V*_OC_ of 0.87 V, *J*_SC_ of 18.44 mA cm^−2^, and an FF of 0.63 which is higher than those of the flexible PEIE‐based device which possesses a PCE of 7.20% with a *V*_OC_ of 0.84 V, *J*_SC_ of 18.21 mA cm^−2^, and FF of 0.49. Different from the rigid OPV devices, the flexible ZnO‐based device shows poor performance with a PCE of only 6.42% with a *V*_OC_ of 0.87 V, *J*_SC_ of 17.75, and FF of 0.42. The EQE spectra of flexible OPV devices are shown in Figure 4e. Notably, the PCE ratio between the rigid (12.06%) and the flexible device (10.11%) with the PEIE‐EDT CIL was 83.83% which was one of the highest compared to those of ZnO and PEIE which have *R* to *F* ratio of 34.59 and 75.00%, respectively. The high PCE ratio between rigid and flexible devices is beneficial for flexible device development.

The bending test was performed to investigate the mechanical capability of flexible OPV devices. The devices were bent with various bending radius and the bending cycles were performed up to 200 times. The flexible PEIE‐EDT‐based devices can retain 98.18% after they were bent with a bending radius of 0.5 cm which is superior to flexible PEIE‐based devices which only can retain 78.48% from initial efficiency (Figure 4f). The better bending stability of PEIE‐EDT is due to its improved contact affinity with the active layer which is proven by a higher contact angle. For flexible ZnO‐based devices, the efficiency drastically decreases after it was bent with a bending radius of 0.75 cm, and the device seems “broken” at a bending radius of 0.5 cm. This declined efficiency of flexible ZnO‐based devices might be associated with the poor mechanical capability of ZnO due to its rigid contact nature.^[^ ^33^ ^]^

After successfully demonstrating the recognizable results of PEIE‐EDT using PBDB‐T:ITIC‐M system, we further tested the performance in different active layers to check the universality of our method in NF systems. The photovoltaic parameters of optimized devices with different active layers are summarized in Table S2 in the Supporting Information and the *J*–*V* curves are shown in Figure S14 in the Supporting Information. As a result, the PEIE‐EDT used as CILs in flexible devices with PCE10:IEICO‐4F and PM6:ITIC‐4F layer performs well with a PCE of 8.85% and 10.11%, respectively. Meanwhile, the flexible devices using PEIE show poor performance with a PCE of 7.39% for PCE10:IEICO‐4F and 4.81% for PM6:ITIC‐4F active layers. From the results, it can be seen that our strategy exhibits universality and can be applied to any NFAs.

### 2.4 Electrical Properties and Recombination Analysis

**Figure 5:**
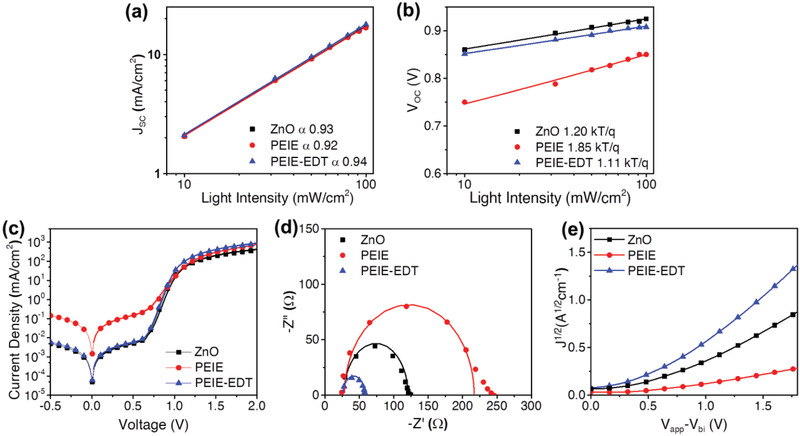
Light intensity as a function of a) *J*_SC_, b) *V*_OCa_; c) semi‐log *J*–*V* curve measured under dark condition; d) Nyquist plot of rigid OPV devices with various CILs measured under 1 sun illumination; e) the SCLC of OPV devices with various CILs.

The *J*_SC_ as a function of the light intensity (**Figure** **5**a) with a power‐law relationship of *J*_SC_ ∝ *I^a^* were fitted to obtain a value of 0.93, 0.92, and 0.94 for ZnO, PEIE, and PEIE‐EDT devices, respectively.^[^ ^34^ ^]^ The *V*_OC_ as a function of the light intensity can be used to distinguish the dominating recombination mechanism, whether trap‐assisted recombination or bimolecular recombination. Either leakage currents or trap‐assisted recombination could alter the slope of the *V*_OC_ as a function of light intensity to become higher than *kT*/*q*.^[^ ^34^, ^35^ ^]^ As shown in Figure 5b, the PEIE device with high leakage currents shows a slope of 1.85 *kT*/*q* indicating the domination of trap‐assisted recombination. Meanwhile, PEIE‐EDT with a slope of 1.11 *kT*/*q* has less dependency on the light intensity indicating the suppressed trap‐assisted recombination.^[^ ^34^, ^36^, ^37^ ^]^

To discuss the effect of EDT treatment on electrical properties and recombination characteristics at the PEIE/active layer interface, the *J*–*V* curve measured under dark conditions is plotted on a semi‐logarithmic scale. Figure 5c represents the *J*–*V* curves with various interfacial layers measured in the dark to evaluate device characteristics such as shunt resistance (*R*_SH_), ideality factor (*n*), and series resistance (*R*_S_) giving physical meanings of leakage currents, device recombination, and bulk resistance, respectively.^[^ ^34^, ^38^, ^39^ ^]^ In this study, the dark *J*–*V* curves were measured by sweeping voltage from −0.5 to 2.0 V and the fitting of the curve was performed from 0 to 2.0 V. The extracted parameters from dark *J*–*V* were summarized in Table S3 in the Supporting Information. From this curve, it can be seen clearly that the pristine PEIE device shows a high leakage current with an *R*_SH_ value of 3.4 × 10^3^ Ω cm^2^ indicating higher charge carrier recombination which is responsible for its low *V*_OC_.^[^ ^34^, ^39^, ^40^ ^]^ However, the leakage current can be significantly suppressed on the PEIE‐EDT‐based device with *R*_SH_ value of 7.2 × 10^4^ Ω cm^2^. We also analyzed the slope of the exponential region of the dark *J*–*V* curves to distinguish the recombination mechanism in the devices. The curves with various interfacial layers show an exponential region at the range of 0.6–1.2 V. The ideality factor of ZnO, PEIE, and PEIE‐EDT devices are 1.46, 1,80, and 1.07 *kT*/*q*. The ideality factor closes to slope *kT*/*q* represents a device with less trap‐assisted recombination as shown by the PEIE‐EDT‐based device.^[^ ^38^, ^41^ ^]^ Here, we assume that the increase in the ideality factor and leakage current obtained from dark *J*–*V* measurement is attributed to trap‐assisted recombination in the “poisoned” ITIC‐M recombination centers.

The electrochemical impedance spectroscopy (EIS) was used to further investigate the electrical characteristics by analyzing differential resistances and capacitance in the devices.^[^ ^42^ ^]^ The semi‐circle curves were fitted using the equivalent circuit as an inset in Figure 5d and the extracted parameters are summarized in Table S4 in the Supporting Information. Series resistance (*R*_S_) represents the resistive losses in the electrodes and bulk resistance in the active layer. The charge transfer resistance (*R*_CT_) represents an aggregate of interfacial resistance between the active layer and cathode interfacial layers, and donor/acceptor interfacial resistance. The capacitance phase element (CPE) illustrates the interface charge transport process and the nonideal capacitor behavior.^[^ ^42^ ^]^ The CPE‐T and CPE‐P are subsequently attributed to the applied amplitude and the degree of ideal behavior. The ZnO, PEIE, and PEIE‐EDT devices show similar *R*_S_ values of 28.28, 28.54, and 27.31 Ω, respectively. The PEIE device exhibits a high *R*_CT_ value of 196 Ω compared to other devices. However, the *R*_CT_ value drastically decreases to 34.90 Ω after the introduction of EDT at the PEIE/active layer interface. The high charge carrier resistance on the PEIE device might be attributed to the interrupted intermolecular charge transport in the ITIC‐M acceptor due to an unwanted reaction between PEIE and ITIC‐M acceptor. After the introduction of EDT, this reaction can be prevented, and the interrupted charge transport is not likely to happen due to the ITIC‐M acceptor in “pure” condition proven by lower charge transport resistance.

The space carrier limited current (SCLC) method was used to determine electron mobility. Electron‐only devices with the structure of ITO/CILs/PBDB‐T:ITIC‐M/LiF/Al was fabricated and measured under dark condition (Figure 5e). The electron mobility calculation was performed using the Mott–Gurney equation: *J* = (9/8)*ε*_0_ *ε*_r_ *μ*(*V* ^2^/*d* ^3^), where the *J* is the current density, *V* is *V*_applied_ − *V*_bi_, *ε*_0_ is the permittivity of free space, *ε*_r_ is the relative dielectric constant assumed to be 3 for our active polymer layer, *d* is the active layer thickness (*d* = 85 nm), and *μ* is the carrier mobility.^[^ ^11^, ^43^ ^]^ The fitting was performed in the SCLC regime with the *R*‐square value maintained over 0.99. The obtained electron mobility (*μ*_e_) values were 5.72 × 10^−4^, 1.80 × 10^−4^, and 8.89 × 10^−4^ cm^2^ V^−1^ s^−1^ for ZnO, PEIE, and PEIE‐EDT electron‐only devices summarized in Table S5 in the Supporting Information. For the pristine PEIE‐based device, the imperfect molecular structure of ITIC‐M acceptor due to unwanted reaction with PEIE could retard the charge transport leads to lower electron mobility.^[^ ^21^ ^]^ Meanwhile, the introduction of EDT treatment significantly increased the electron mobility by hindering the undesired reaction between PEIE and ITIC resulting in improved charge carrier transport and reduced interfacial resistance in the active layer or at the CIL/active interface.

Under the low light condition, the *V*_OC_ loss becomes one of the substantial issues. However, in this work, the *V*_OC_ loss can be reduced by simply put EDT at the CILs/active layer interface. Figure S15 in the Supporting Information shows the *J*–*V* curve of the ZnO, PEIE, and PEIE‐EDT‐based devices measured under LED 2700K, and the obtained photovoltaic parameters are summarized in Table S6 in the Supporting Information and its bar graph is shown in Figure S16 in the Supporting Information. The *J*_SC_ significantly increases as the increased light intensity from 200 to 500 lux. The *V*_OC_ also is found to increase with the increased light intensity from 200 to 500 lux. If we compare with the photovoltaic properties obtained under 1 sun illumination, the PEIE‐EDT‐based devices exhibit the smallest *V*_OC_ loss compared to PEIE‐based devices. For example, the PEIE‐EDT shows a *V*_OC_ of 0.71 V under 200 lux, which is 0.19 V lower compared to 1 sun measurement while the PEIE‐based device shows *V*_OC_ of 0.62 V, which is 0.24 V lower. Since the shunt resistance strongly influences solar cell performance at low light conditions, we suggest that the higher performance of the PEIE‐EDT‐based device under low light illumination is related to lower leakage current, low voltage loss, and trap‐assisted recombination.^[^ ^34^, ^35^, ^44^ ^]^

## 3 Conclusion

We demonstrated a method that can significantly improve the performance of BHJ OPV by inserting EDT at the PEIE/active layer interface. This method can mitigate the reaction that usually occurs between PEIE and NFA. We strongly realize that the superiority of our strategy is based on two aspects. First, the insertion of EDT to PEIE/active layer interface does not require additional annealing treatment that will benefit for flexible device fabrication. Second, besides mitigating the reaction between PEIE and NFA, the insertion of EDT also can lower the WF by improving electron flow toward ITO direction. We also found that the improved parameters, especially *V*_OC_, after EDT treatment on PEIE are mainly related to the reduced leakage currents, decreased WF, and decreased trap‐assisted recombination as well as lower electron mobility. This study will become an important development in flexible photovoltaics, especially the OPV with the NFA, where the reactions between amine‐rich polyelectrolytes become a huge “stumbling block” for its development.

## 4 Experimental Section

### Materials

Poly[(2,6‐(4,8‐bis(5‐(2‐ethylhexyl)thiophen‐2‐yl)‐benzo[1,2‐*b*:4,5‐*b*’]dithiophene))‐alt‐(5,5‐(1’,3’‐di‐2‐thienyl‐5’,7’‐bis(2‐ethylhexyl)benzo[1’,2’‐*c*:4’,5’‐*c*’]dithiophene‐4,8‐dione)] (PBDB‐T), 3,9‐bis(2‐methylene‐((3‐(1,1‐dicyanomethylene)‐6/7‐methyl)‐indanone))‐5,5,11,11‐tetrakis(4‐hexylphenyl)‐dithieno[2,3‐*d*:2’,3’‐*d*’]‐*s*‐indaceno[1,2‐*b*:5,6‐*b*’]dithiophene (ITIC‐M) and 1,2 ethanedithiol (Merck), and acetonitrile (Merck) were purchased commercially. Chlorobenzene (CB), 1,8‐diidooctane (DIO), polyethylenimine 80% ethoxylated solution, and methanol were purchased from Sigma Aldrich.

### Device Fabrication

ITO glass substrates were sonicated using ultrasonic baths in acetone and isopropanol for 10 min and dried at 100 °C for 1 h in the oven afterward. For a flexible device, the polyethylene naphthalate substrates were washed by methanol using swabs. Pre‐cleaned ITO substrates were treated using UV‐ozone treatment for 17 min. PEIE solution was prepared by dissolving the 0.05 wt% of polyethyleneimine ethoxylated in methanol followed by stirring for 1 h at room temperature. The PEIE solution was spin‐coated on the ITO substrate at 2000 rpm for 60 s and dried in an Ar‐filled glove‐box at room temperature for 15 min. The EDT solution was prepared by dissolving the 1,2‐ethanedithiol in acetonitrile with a concentration of 1 × 10^−2^ m. For PEIE‐EDT device, PEIE‐coated ITO was soaked in the EDT solution for 60 s, followed by spin‐coating at 3000 rpm for 40 s. To remove excess EDT on the PEIE surface, the acetonitrile was used for rinsing by spin‐coating it at 3000 rpm for 40 s. For comparison, the ZnO nanoparticles were spin‐coated on the ITO substrate at 3000 rpm for 40 s. The blend active solution PBDB‐T:ITIC‐M (10 mg:10 mg) dissolved in 992.5 µL of CB and 7.5 µL of DIO was spin‐coated on the samples at 3000 rpm for 60 s. The devices were annealed at 100 °C for 5 min before thermal deposition. For comparison of active layers, a mixture of PM6:ITIC‐4F (10:12 mg) dissolved in 1 mL of CB with 0.75% DIO and or PTB7‐th:IEICO‐4F (10:15 mg) dissolved in 1 mL of CB with 0.5% CN was spin‐coated on the CILs. The 7 nm of MoOx and 100 nm of Ag electrodes were thermally deposited using a thermal evaporator at high‐vacuum condition (<3 × 10^−6^ Torr) with the area active 0.125 cm^2^.

### Measurements and Characterizations

The *J*–*V* measurement was carried out using a Keithley 2400A source unit under and calibrated AM 1.5G simulated illumination of 100 mW cm^−2^. For low light *J*–*V* measurement, the LED 2700K light source was used. EQE or incident photon‐to‐electron conversion efficiency (IPCE) spectra were measured from a wavelength of 300–800 nm using the EQE measurement system (Newport Co., Oriel IQE‐200 system). The optical transmittance and absorbance spectra of thin films were measured using a UV‐vis spectrometer (Varian, Cary5000). The work functions of PEIE and PEIE‐EDT layers on ITO substrates were measured by using UPS with a He I source (21.2 eV). For the XPS measurement, the samples were prepared on ITO glass substrates and measured by using X‐ray Photoelectron Spectrometer Multi‐lab 2000. The EIS measurement was performed by using an AC impedance analyzer compactstat electrochemical interface (IVIUM Technologies) in a frequency range of 100–1 MHz and the applied bias voltage was set close to *V*_OC_. The EIS measurement was done under AM 1.5G one sun illumination. C‐AFM‐based *I*–*V* measurement was carried out at the contact mode by applying a DC voltage between Pt/Ir‐coated cantilever tip and a bottom electrode. Pt/Ir‐coated Si cantilever with a spring constant of 0.2 N m^−1^ was used to mapping a conductive area. At first, a 5 µm × 5 µm area scanning was carried out with −0.5 and 0.5 V bias to verify the conducting status of photovoltaics. The topology and current images were simultaneously obtained. In the current image, the bright area represented a high conductance while the dark area indicated a low conductance in the case of 0.5 V bias applied. On the other hand, −0.5 V bias was applied, it was the opposite of the former case. The FTIR spectra were obtained by using Perkin Elmer Spectrum 100. The ^1^H NMR spectra were collected in a CD_2_Cl_2_ reagent.

### Statistical Analysis

The obtained FF was transformed from percent format to decimal. Data were presented as mean ± SD, the sample number *n* = 12 for each device. The data distribution was presented using the normal curve. All statistical analysis was performed using the Origin 2019b.

## Conflict of Interest

The authors declare no conflict of interest.

## Supporting information

Supporting Information

## Data Availability

Research data are not shared.
