# Supplementary material for: Mitigating the Undesirable Chemical Reaction between Organic Molecules for Highly Efficient Flexible Organic Photovoltaics
Source: Adv Sci (Weinh). 2021 May 7;8(14):2100865. doi: 10.1002/advs.202100865 (PMC8292892; doi:10.1002/advs.202100865)
Supplement: Supplementary file 1 — Supporting Information [file ADVS-8-2100865-s001.pdf]

© 2021 Wiley-VCH GmbH

ADVANCED SCIENCE
Open Access

Supporting Information

for *Adv. Sci.*, DOI: 10.1002/advs.202100865

Mitigating the Undesirable Chemical Reaction between Organic Molecules for Highly Efficient Flexible Organic Photovoltaics

*Adi Prasetio, Muhammad Jahandar, Soyeon Kim, Jinhee Heo, Yong Hyun Kim, and Dong Chan Lim**

((Supporting Information can be included here using this template))

Supporting Information

Mitigating the undesirable chemical reaction between organic molecules for highly efficient flexible organic photovoltaics

*Adi Prasetio, Muhammad Jahandar, Soyeon Kim, Jinhee Heo, Yong Hyun Kim, and Dong Chan Lim**

1. Supplementary Figures

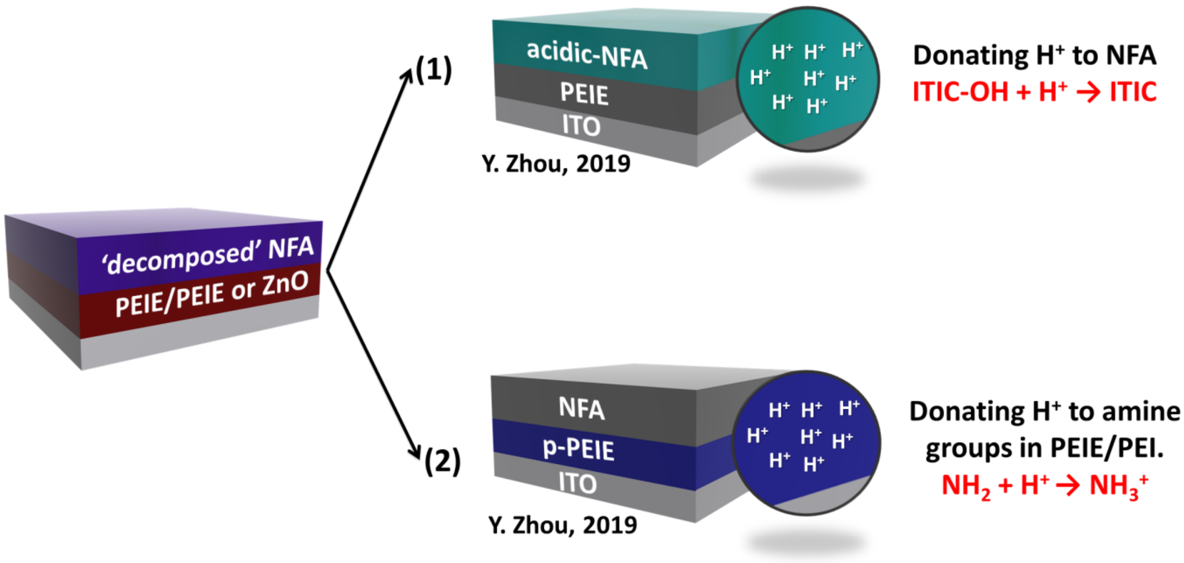

Figure S1 The schematic of the various strategies to mitigate reaction occurred in NF acceptor when contact with amines- or hydroxyl-groups in interfacial layers. The 'Poisoned' NF acceptor due to reaction with the PEIE/PEI interfacial layer or with ZnO nps after UV-light exposure can be prevented by (1) protonated-PEIE by adding the H+ to PEIE/PEI, (2) 'acidic' environment by directly adding H+ to NF acceptor.

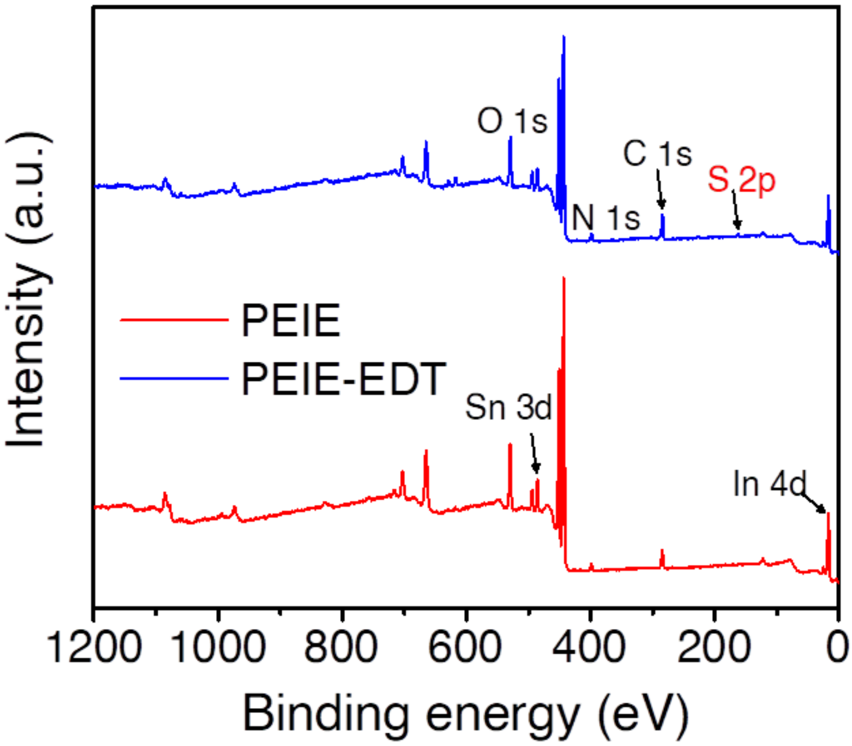

Figure S2 the X-ray photoelectron spectroscopy (XPS) spectra of PEIE and PEIE-EDT thin films on ITO glass substrates.

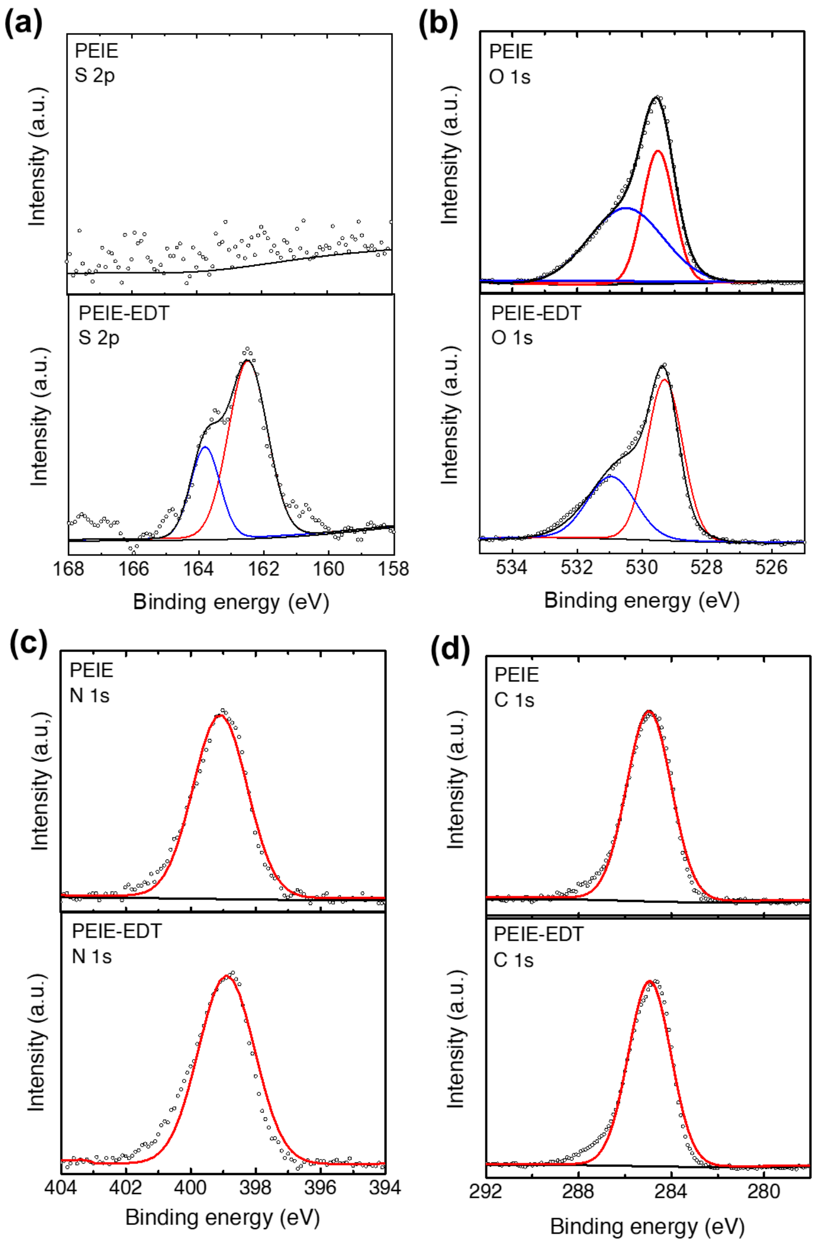

Figure S3 XPS analysis results of PEIE and PEIE-EDT with specific spectra of (a) S 2p, (b) O 1s, (c) N 1s, and (d) C 1s.

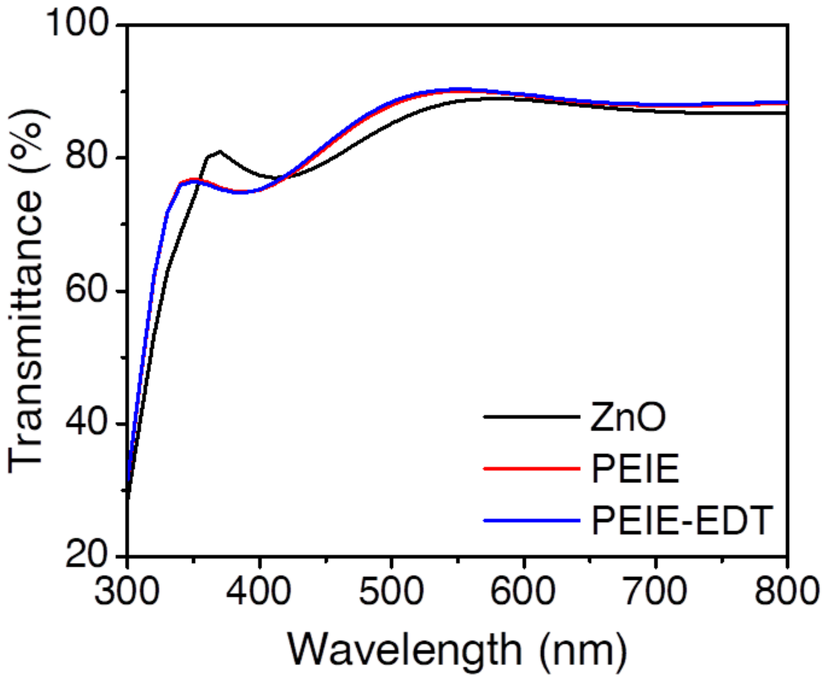

Figure S4 Transmittance of ZnO, PEIE and PEIE with EDT on ITO glass substrates.

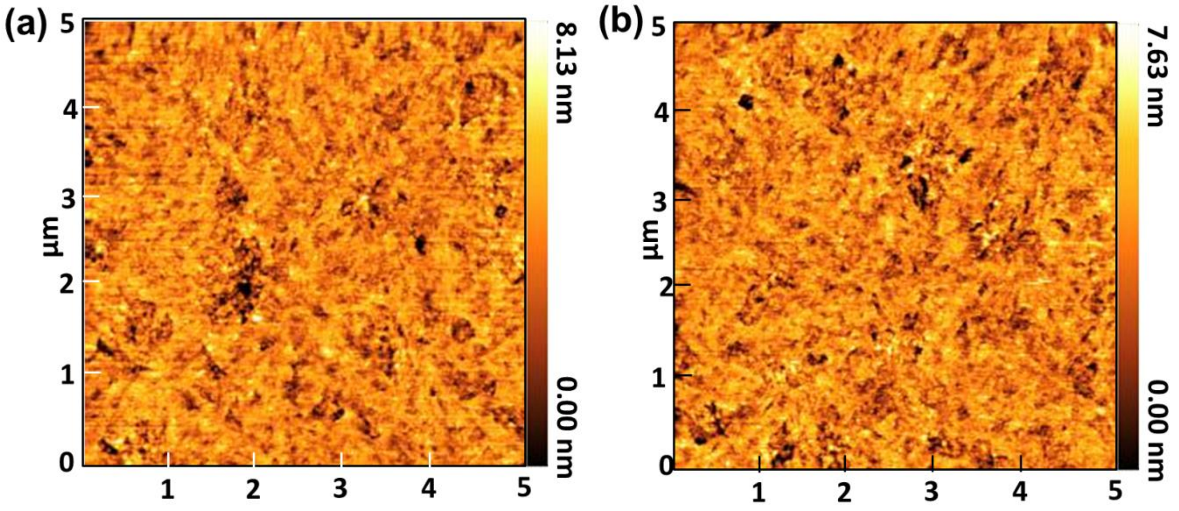

Figure S5 The atomic force microspcopy (AFM) image of (a) PEIE and (b) PEIE-EDT films on ITO glass susbtrates.

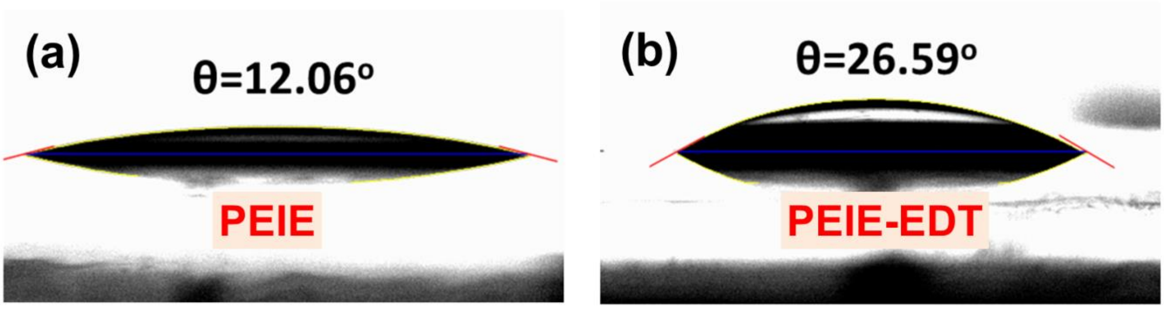

Figure S6 Contact angle of (a) PEIE, and (b) PEIE-EDT with DI water drop.

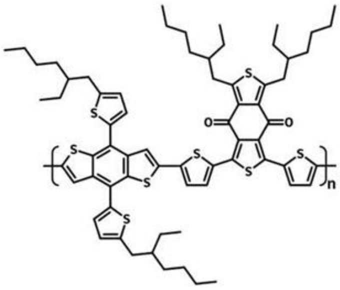

PBDB-T

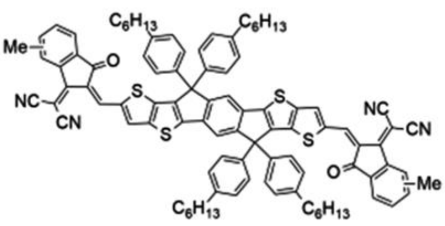

ITIC-M

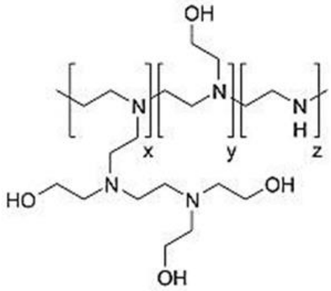

PEIE

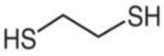

1,2 Ethanedithiol

Figure S7 The chemical structure of chemical used in this study.

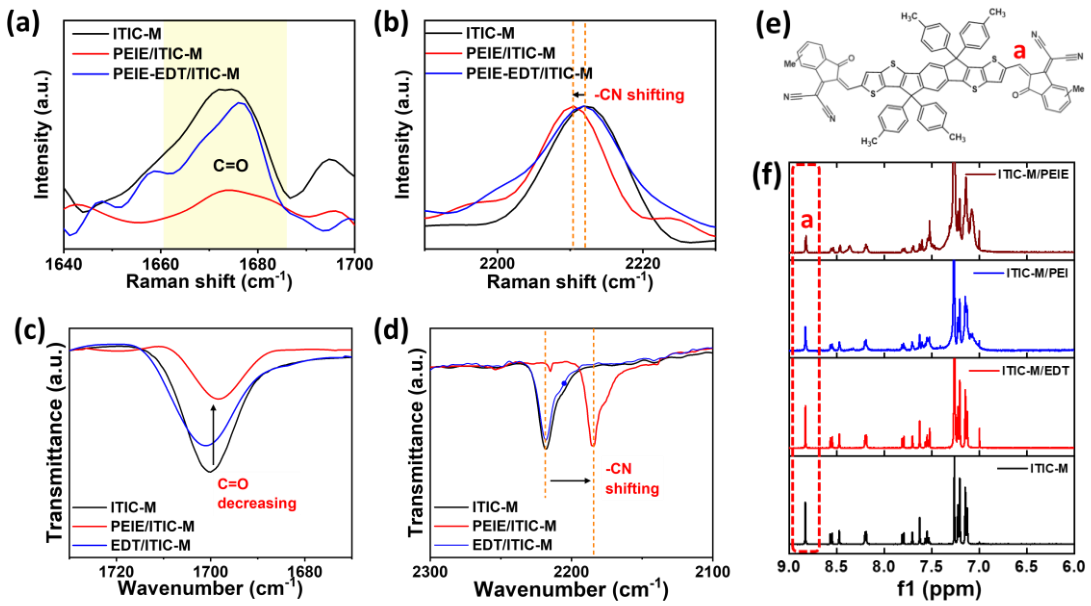

Figure S8 Raman spectra of (a) C=O, and (b) -CN groups for ITIC-M coated on bare ITO (labeled as ITIC-M), PEIE (labeled as PEIE/ITIC-M) and PEIE-EDT (labeled as PEIE-EDT/ITIC-M); FTIR spectra of (c) C=O, and (d) -CN groups for ITIC-M and ITIC-M mixed with PEIE and EDT; (e) The chemical structure of ITIC-M marked with “a“ and (f) 1H NMR spectra of pristine ITIC-M and ITIC-M after adding EDT, PEI, and PEIE. The marked dash box is for showing the intensity change of H at the position of marked “a” in the chemical structure.

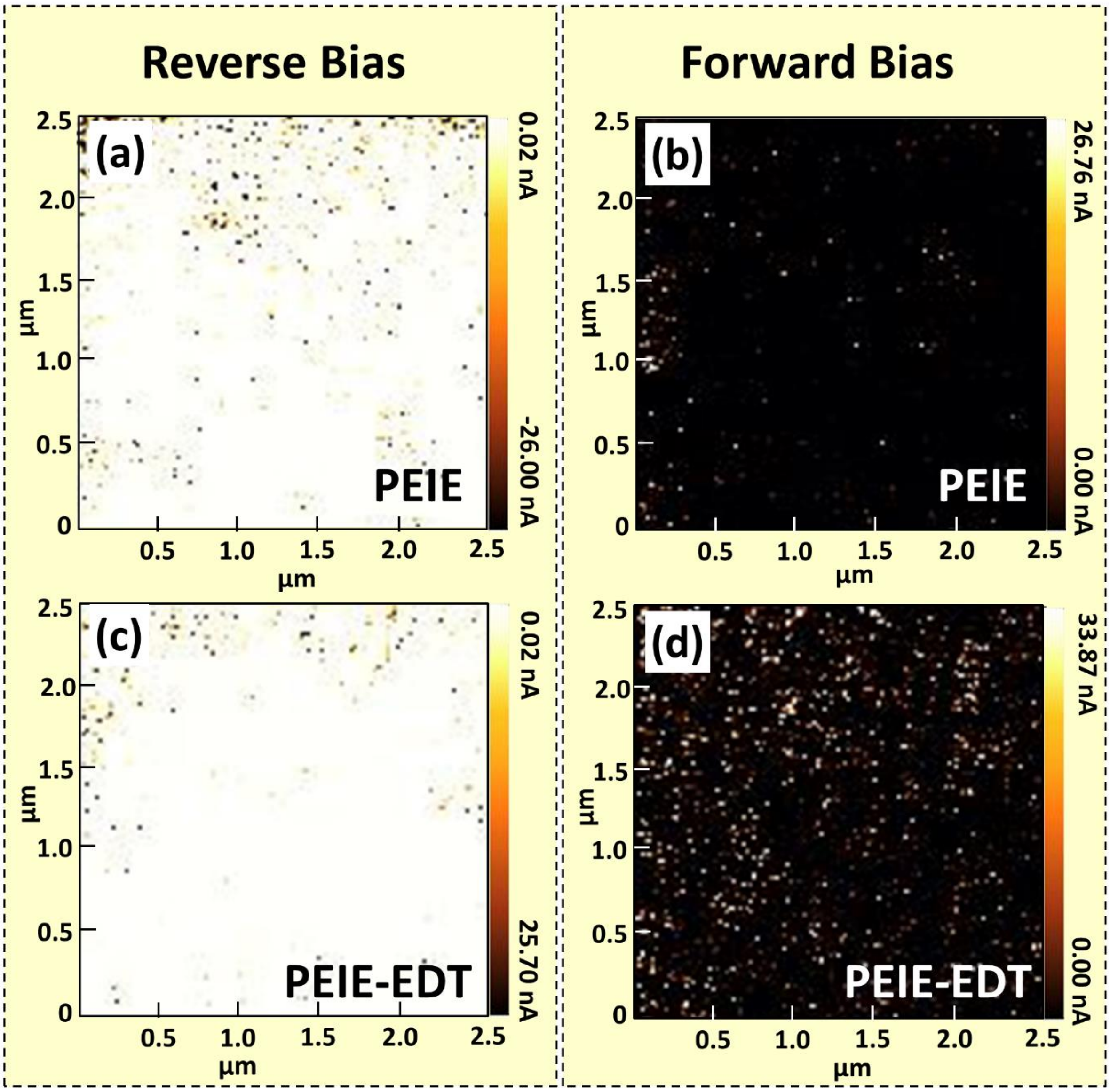

Figure S9 The conductive-AFM (C-AFM) image of (a) PEIE under reverse bias, (b) PEIE under forward bias, (c) PEIE-EDT under reverse bias, and (d) PEIE-EDT under forward bias. In the reverse bias, the darkest part refers to the highest current. Meanwhile, in the forward bias, the most brightness part refers to the highest current.

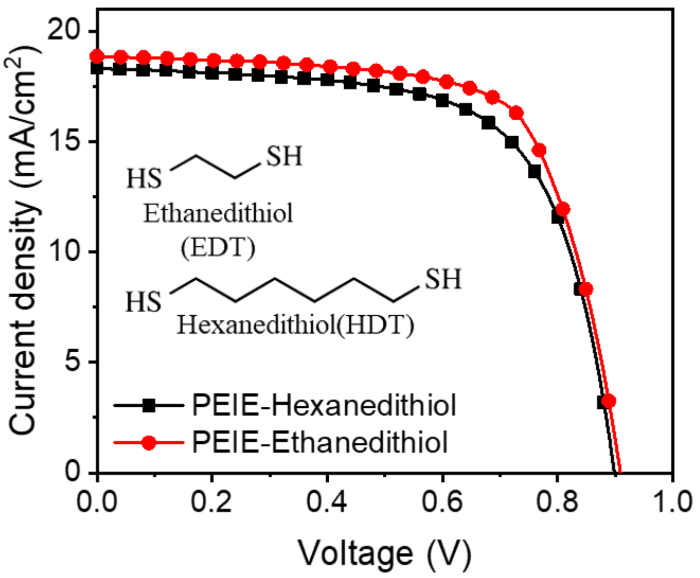

Figure S10 (a) The J-V curve of the rigid OPV with ethanedithiol and hexanedithiol on PEIE layer.

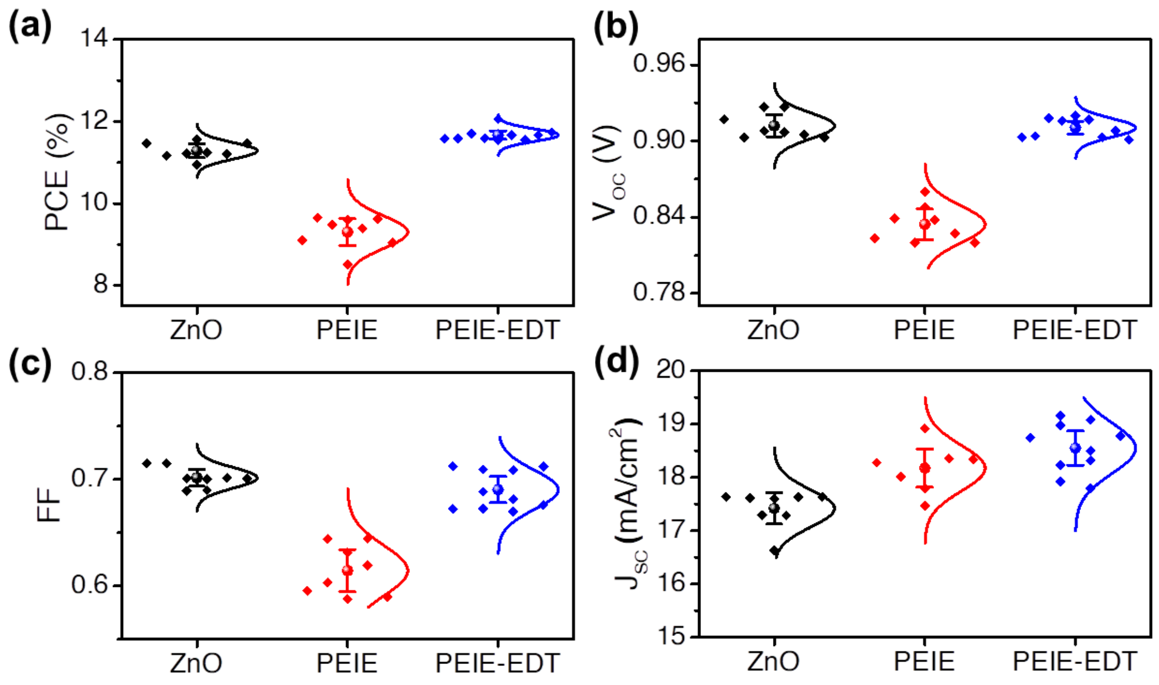

Figure S11 Device statistics of (a) PCE, (b) V_{OC}, (c) FF, and (d) J_{SC} for rigid OPV devices with various CILs.

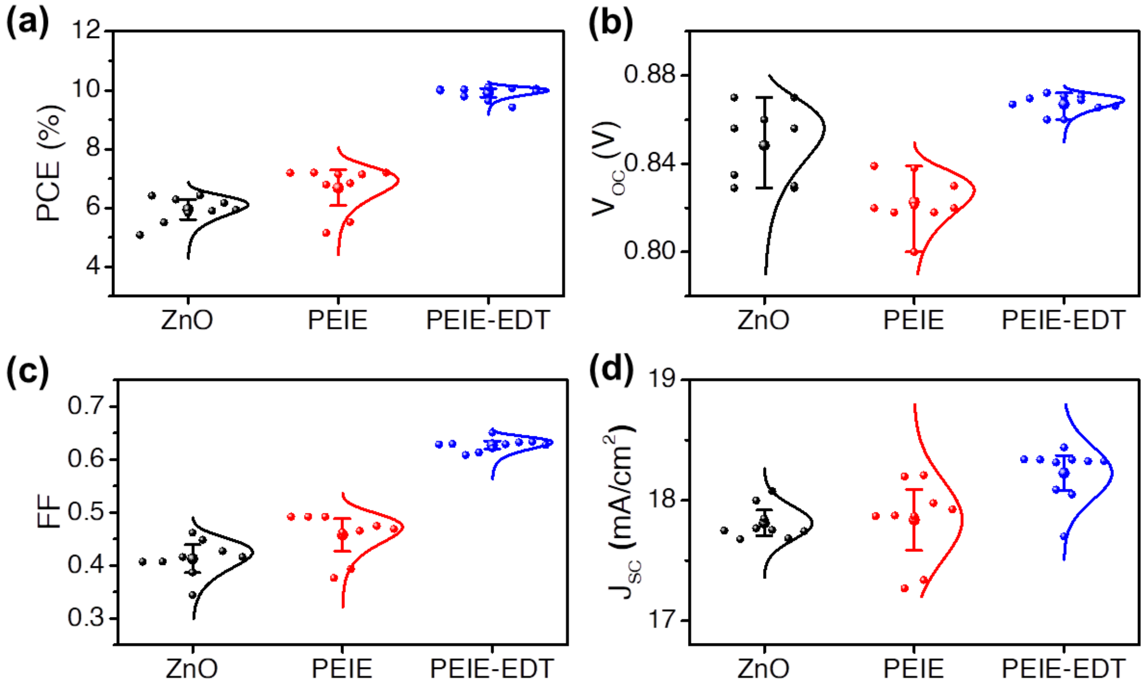

Figure S12 Device statistics of (a) PCE, (b) V_{OC}, (c) FF, and (d) J_{SC} for flexible OPV devices with various CILs.

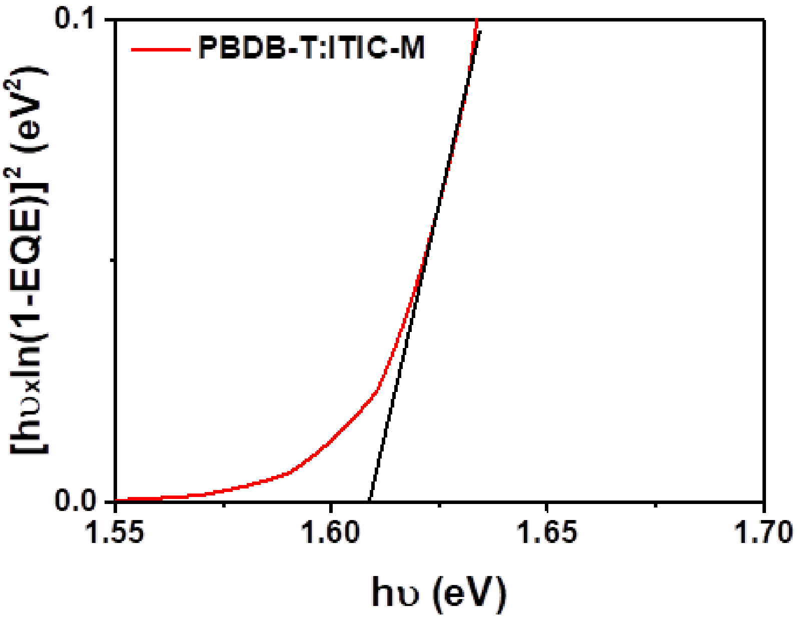

Figure S13 Tauq plot for determining energy gap of PBDB-T:ITIC-M active layer.

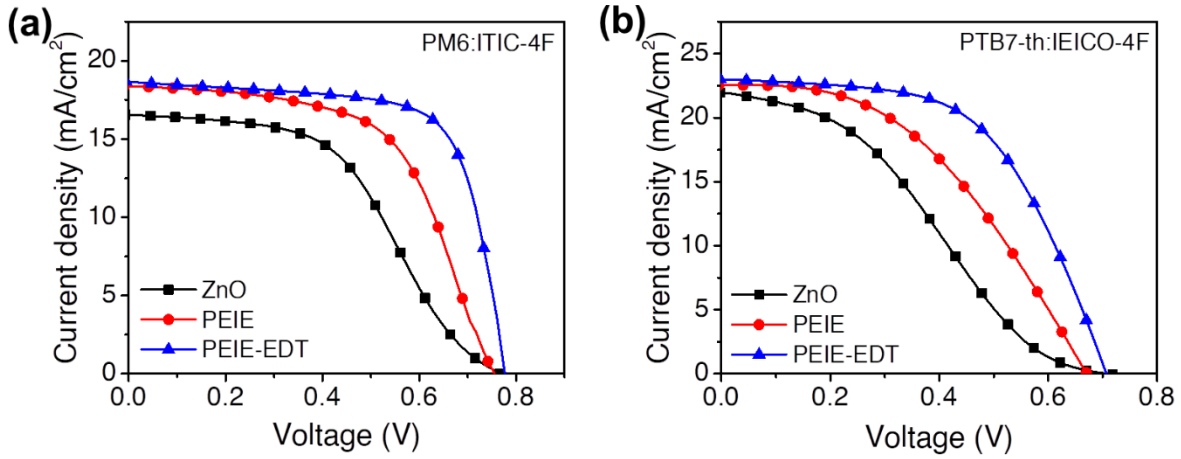

Figure S14. J-V curve of flexible devices with (a) PM6:ITIC-4F active layer, and (b) PTB7-th:IEICO-4F.

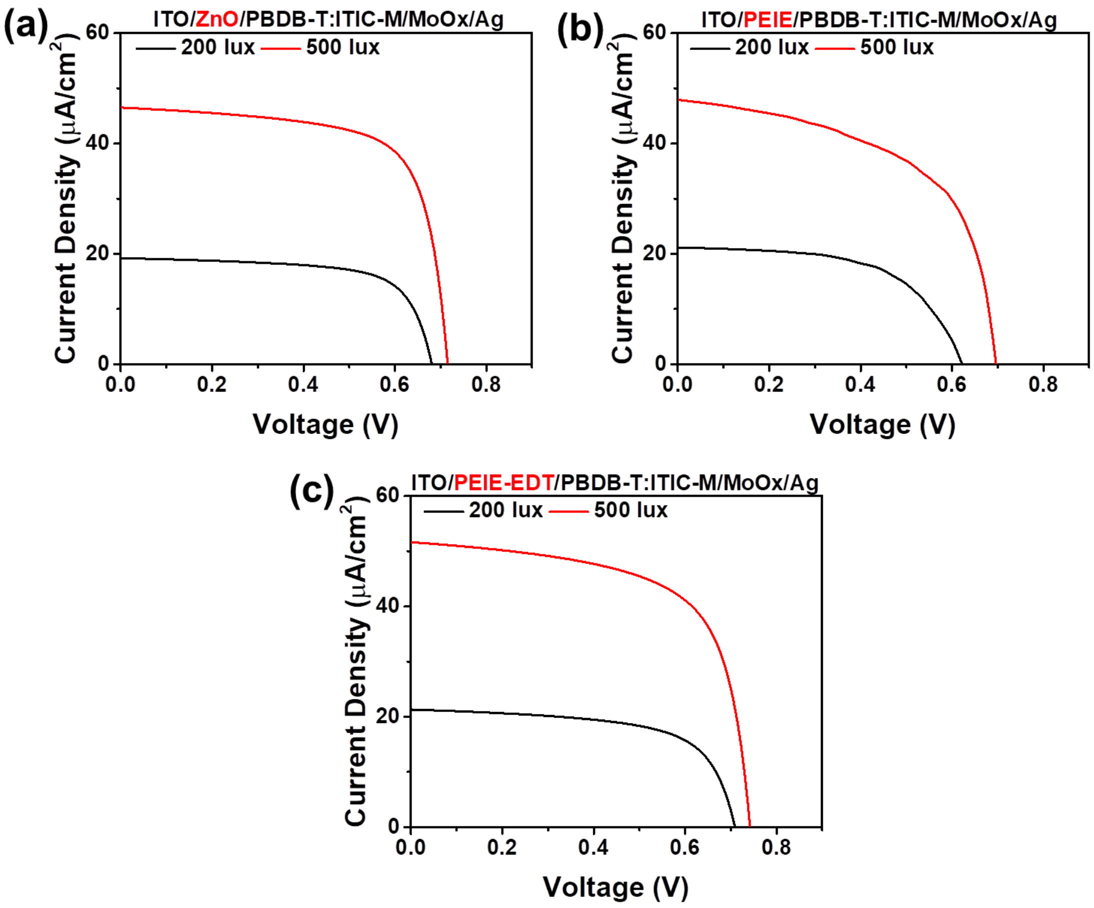

Figure S15. The J-V curve of the rigid OPVs based on PBDB-T:ITIC-M photoactive layer with (a) ZnO, (b) PEIE, and (c) PEIE-EDT CILs measured under LED 2700K illumination with the light intensity of 200 and 500 lux.

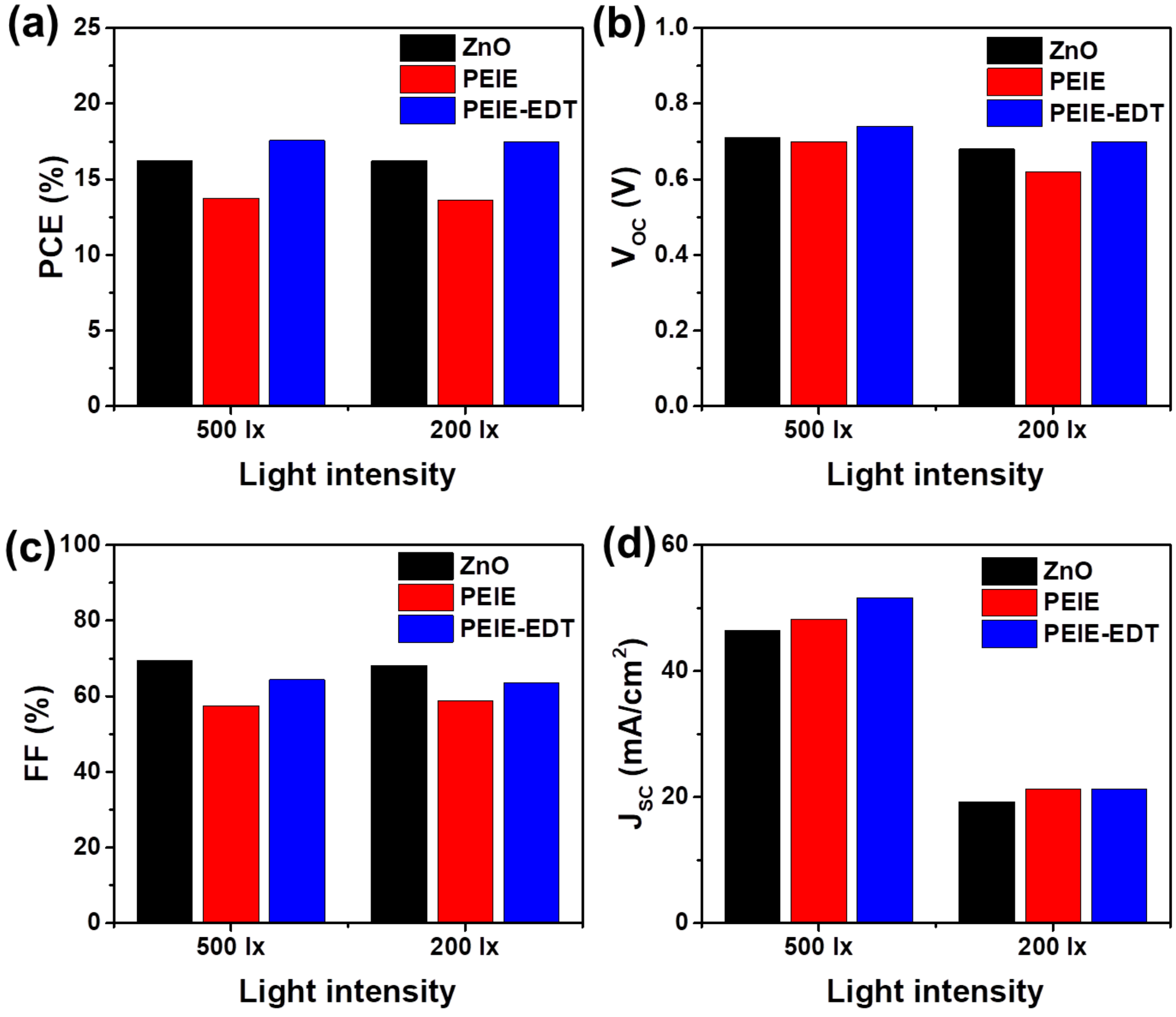

Figure S16. Bar graphs showing the photovoltaic properties of (a) PCE, (b) J_{SC}, (c) V_{OC}, and (d) FF of rigid OPV devices measured under LED 2700K illumination with the light intensity of 200 and 500 lux.

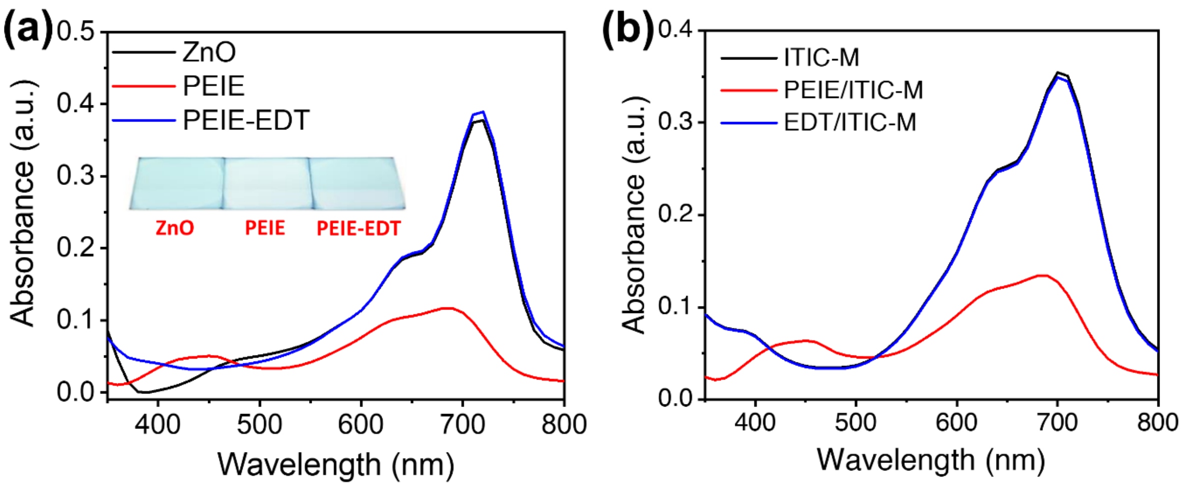

Figure S17. UV-Vis absorbance spectra of (a) ITIC-M film coated on ZnO, PEIE and PEIE-EDT, (b) ITIC-M, and ITIC-M exposed to PEIE and EDT.

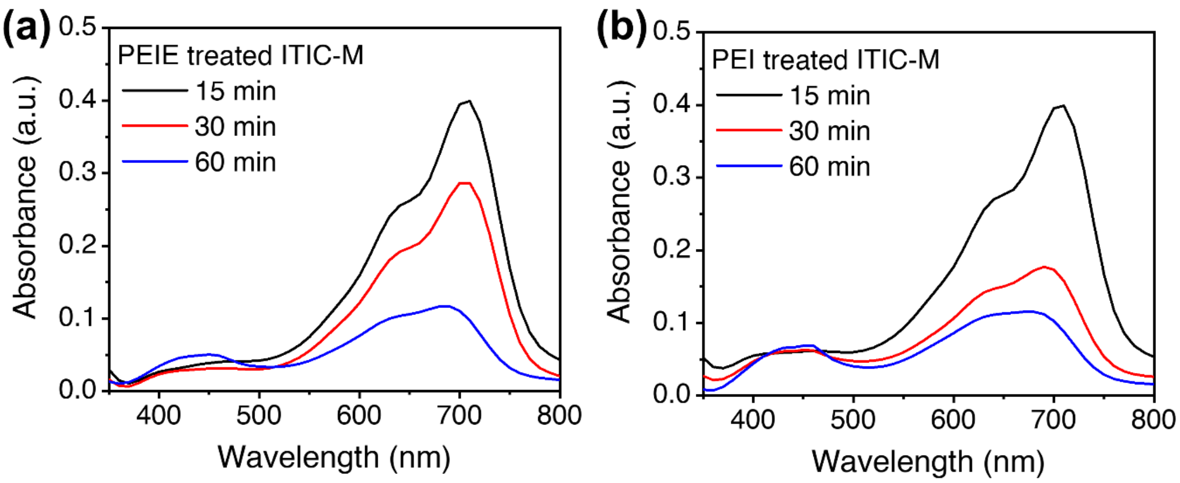

Figure S18. UV-Vis absorbance spectra of ITIC-M film exposed to (a) PEIE and (b) PEI with different exposure duration.

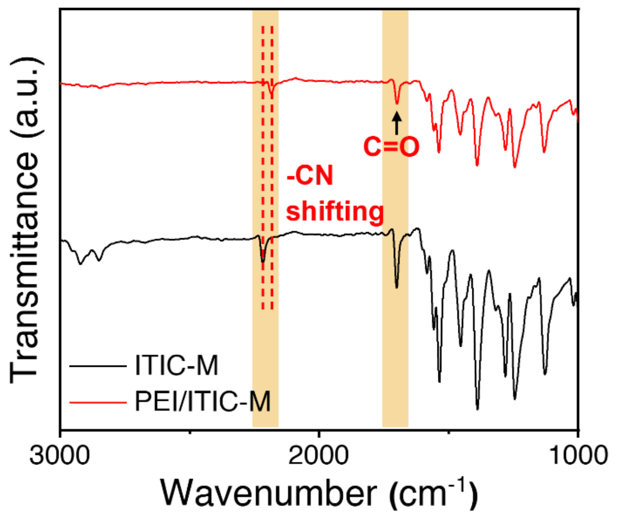

Figure S19. FTIR spectra of ITIC-M and ITIC-M exposed to PEI.

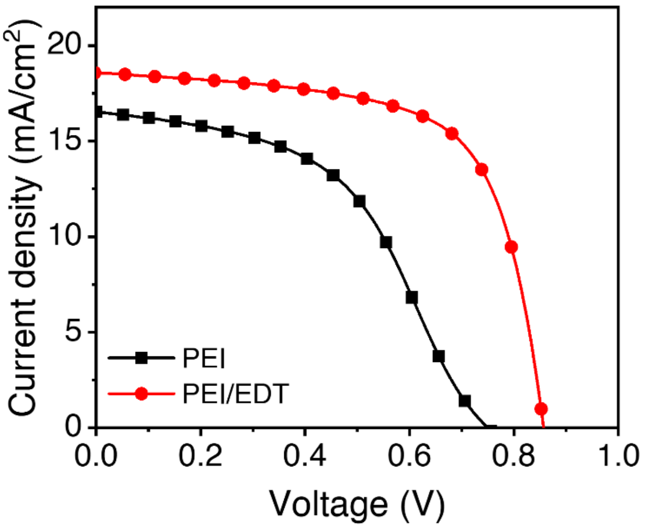

Figure S20. Current-voltage (J-V) curve of PBDB-T:ITIC-M based OPV devices with PEI and PEI-EDT CILs.

2. Supplementary Tables

Table S1. Photovoltaic properties of rigid OPV devices with ethanedithiol (EDT) and hexanedithiol (HDT) on PEIE layer.

CIL	PCE (%)	V_{OC} (V)	J_{SC} (mA/cm^2)	FF
PEIE-EDT	11.90	0.91	18.85	0.69
PEIE-HDT	10.82	0.89	18.32	0.66

Table S2. Photovoltaic properties of flexible OPV devices with various active layer and CILs.

Active layer	CILs	PCE (%)	V_{OC} (V)	J_{SC} (mA/cm^2)	FF
PCE10:IEICO-4F	ZnO	4.86	0.77	16.42	0.38
	PEIE	7.39	0.66	22.84	0.49
	PEIE-EDT	8.85	0.70	23.01	0.54
PM6:IT-4F	ZnO	6.05	0.77	16.543	0.48
	PEIE	4.81	0.61	15.98	0.49
	PEIE-EDT	10.11	0.77	18.86	0.70

Table S3 Parameters extracted from the fitting of the dark J-V curve

CILs	R_{SH} (Ω)	R_S (Ω)	n
ZnO	$7.5x10^4$	1.99	1.46
PEIE	$3.4x10^3$	1.53	1.80
PEIE-EDT	$7.2x10^4$	1.20	1.07

Table S4: Parameters employed for the fitting of the impedance spectra by use of an equivalent circuit model.

CILs	R_S (Ω)	R_{CT} (Ω)	CPE-T (F/cm^2)	CPE-P
ZnO	28.28	91.84	$3.47x10^{-8}$	0.97
PEIE	28.54	196.00	$4.10x10^{-8}$	0.93
PEIE-EDT	27.31	34.90	$5.19x10^{-8}$	0.98

Table S5: Electron mobility extracted from the SCLC fitting.

CILs	μe (cm^2 V^{-1} s^{-1})
ZnO	$5.72x10^{-4}$
PEIE	$1.80x10^{-4}$
PEIE-EDT	$8.89x10^{-4}$

Table S6 The detailed device performance parameters of the rigid OPVs based on PBDB-T:ITIC-M photoactive layer with ZnO, PEIE and PEIE-EDT CILs measured under LED 2700K illumination with the light intensity of 200 and 500 lux.

Device	Intensity (lux)	Pin (μW/cm^2)	V_{OC} (V)	J_{SC} (μA/cm^2)	FF	Pout (μW/cm^2)	PCE (%)
ZnO	200	55.55	0.68	19.24	0.682	8.96	16.22
	500	141.20	0.71	46.50	0.695	23.12	16.25
PEIE	200	55.55	0.62	21.25	0.587	7.76	13.63
	500	141.20	0.70	48.25	0.575	18.88	13.74
PEIE-EDT	200	55.55	0.71	21.31	0.635	9.60	17.48
	500	141.20	0.74	51.62	0.644	24.64	17.57

Table S7. Photovoltaic properties of rigid PBDB-T:ITIC-M based OPV devices with PEI and PEI-EDT CILs.

CIL	PCE (%)	V_{OC} (V)	J_{SC} (mA/cm^2)	FF
PEI	6.04	0.75	16.53	0.49
PEI-EDT	10.45	0.86	18.57	0.68
